# Collagen type I alters the proteomic signature of macrophages in a collagen morphology-dependent manner

**DOI:** 10.1038/s41598-023-32715-0

**Published:** 2023-04-06

**Authors:** Gwenda F. Vasse, Sara Russo, Andrei Barcaru, Asmaa A. A. Oun, Amalia M. Dolga, Patrick van Rijn, Marcel Kwiatkowski, Natalia Govorukhina, Rainer Bischoff, Barbro N. Melgert

**Affiliations:** 1 Biomedical Engineering Department-FB40, University Medical Center Groningen, University of Groningen, Groningen, The Netherlands; 2 University Medical Center Groningen, W.J. Kolff Institute for Biomedical Engineering and Materials Science-FB41, University of Groningen, Groningen, The Netherlands; 3 Department of Molecular Pharmacology, Groningen Research Institute of Pharmacy, University of Groningen, Groningen, The Netherlands; 4 University Medical Center Groningen, Groningen Research Institute for Asthma and COPD (GRIAC), University of Groningen, Groningen, The Netherlands; 5 Department of Analytical Biochemistry, Groningen Research Institute of Pharmacy, University of Groningen, Groningen, The Netherlands; 6 Department of Laboratory Medicine, University Medical Center Groningen, University of Groningen, Groningen, The Netherlands; 7 Department of Cell Biochemistry, Groningen Institute of Biomolecular Sciences & Biotechnology, University of Groningen, Groningen, The Netherlands; 8 Functional Proteo-Metabolomics, Department of Biochemistry, University of Innsbruck, Innsbruck, Austria

**Keywords:** Alveolar macrophages, Respiratory tract diseases, Proteomic analysis

## Abstract

Idiopathic pulmonary fibrosis is a progressive lung disease that causes scarring and loss of lung function. Macrophages play a key role in fibrosis, but their responses to altered morphological and mechanical properties of the extracellular matrix in fibrosis is relatively unexplored. Our previous work showed functional changes in murine fetal liver-derived alveolar macrophages on fibrous or globular collagen morphologies. In this study, we applied differential proteomics to further investigate molecular mechanisms underlying the observed functional changes. Macrophages cultured on uncoated, fibrous, or globular collagen-coated plastic were analyzed by liquid chromatography-mass spectrometry. The presence of collagen affected expression of 77 proteins, while 142 were differentially expressed between macrophages grown on fibrous or globular collagen. Biological process and pathway enrichment analysis revealed that culturing on any type of collagen induced higher expression of enzymes involved in glycolysis. However, this did not lead to a higher rate of glycolysis, probably because of a concomitant decrease in activity of these enzymes. Our data suggest that macrophages sense collagen morphologies and can respond with changes in expression and activity of metabolism-related proteins. These findings suggest intimate interactions between macrophages and their surroundings that may be important in repair or fibrosis of lung tissue.

## Introduction

Fibrosis is defined as excessive accumulation of extracellular matrix (ECM), which eventually impairs the function of an affected organ. Idiopathic pulmonary fibrosis (IPF) is a chronic and progressive lung disease with a short life expectancy of 2–3 years after diagnosis. Treatment options are limited, as only two drugs have been approved that slow down disease progression: nintedanib and pirfenidone^1^.

The characteristic excessive deposition of ECM proteins in fibrosis, mostly by myofibroblasts, changes the biochemical composition of a tissue. However, it is becoming clear that ECM can also be structurally different because of aberrant post-translational modifications^2^. For example, fibrils of collagen type I, the most abundant ECM protein in fibrosis, are structurally different in lung tissue from patients with IPF compared to control lung tissue^3^. All changes in biochemical composition and post-translational modifications also affect the biophysical properties of fibrotic tissue, such as tissue stiffness and topography^4–6^. The resulting biochemically and biophysically altered ECM can be sensed by resident cells and subsequently change their behavior, resulting in constant two-way interactions^7^.

Macrophages are important regulators of ECM homeostasis, as they can stimulate both the production and degradation of ECM proteins^8^. Their ability to prevent or resolve fibrosis in combination with the aberrant macrophage polarization observed in fibrosis, makes studying their possible role in the pathogenesis of fibrosis crucial. Although the effect of fibrosis-related soluble factors (such as cytokines) on macrophage polarization and function has been studied extensively^9,10^, their ability to respond to fibrosis-related morphological changes in the ECM is relatively unexplored^10^.

Previously, we have shown that changes in the morphology of collagen type I, either globular or fibrous, can affect the shape, marker expression and behavior of murine fetal liver-derived alveolar macrophages^11^. Higher expression of the mannose receptor (CD206), known to be upregulated on alveolar macrophages in IPF, was found when alveolar macrophages were cultured on globular collagen. Fibrous collagen led to higher expression of Ym1, a murine marker of pro-healing macrophages. Moreover, macrophage shape changed distinctly in response to fibrous and globular collagen, with a more amoeboid appearance on fibrous collagen and a more mesenchymal appearance with many filopodia on globular collagen. In parallel with these alterations in macrophage shape, transmigration was higher when macrophages were cultured on fibrous collagen compared to the uncoated condition^11^. However, the exact mechanisms behind these characteristic responses are still elusive. In this study, we applied differential proteomics and metabolic analysis to further elucidate macrophage responses to distinct collagen type I morphologies and to unravel possible molecular mechanisms by which the altered ECM in fibrosis affects macrophage function.

## Results

### Proteome analysis

**Figure 1:**
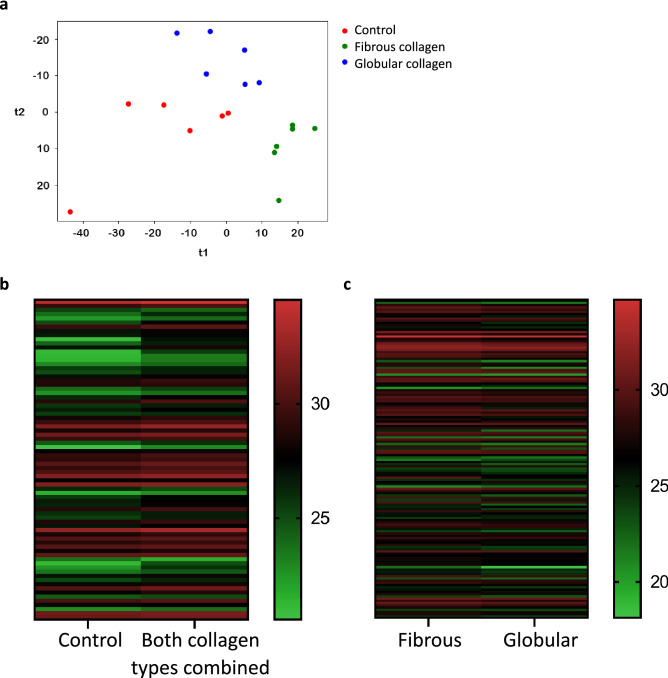
Effect of culturing conditions on the protein expression profile of macrophages. Alveolar macrophages were cultured on uncoated, globular collagen-coated or fibrous collagen-coated tissue culture plastic (n = 6). (**a**) Partial least squares-discriminant analysis plot of alveolar macrophages in the different conditions showing that the 3 conditions cluster separately. (**b**) Heatmap of 77 proteins that were differentially expressed (p < 0.025) between uncoated and collagen type I-coated conditions (fibrous and globular pooled). (**c**) Heatmap of 142 proteins that were differentially expressed (p < 0.05) between macrophages cultured on fibrous or globular collagen. Expressed as Log2 of the median values and statistically tested with an unpaired (**b**) or a paired (**c**) Wilcoxon test.

To investigate the effect of collagen morphology on the proteome signature of macrophages, fetal liver-derived alveolar macrophages were cultured on tissue culture plastic coated with either fibrous or globular collagen, or left uncoated (control) for 72 h. The reason for using this model instead of primary alveolar macrophages was to eliminate any potential interference of transplantation effects that primary alveolar macrophages may experience in cell culture^12^. Primary alveolar macrophages that are removed from their lung-specific environment, which includes exposure to air and cyclic stretch, may experience changes that could affect their responses to collagen sensing. Cell lysates were analyzed by LC–MS in DIA mode. After data processing using our fetal liver-derived alveolar macrophage-specific protein library, 2.870 unique proteins were reproducibly quantified in the three distinct conditions. Partial least squares discriminant analysis indicated that all conditions could be separated based on their proteomic profile (Fig. 1a) after data normalization (Supplementary Fig. 1).

We first investigated the effects of culturing macrophages on collagen versus uncoated plastic. Culturing macrophages on collagen type I-coated tissue culture plastic resulted in differential expression of 77 proteins compared to macrophages on uncoated plastic (Fig. 1b and Supplementary Table 1). 69 of these proteins were more than 1.25-fold upregulated, whereas only three proteins were more than 1.25-fold downregulated (fold change > 0.875) (Supplementary Table 1). To assess collagen morphology-specific effects, we also compared protein expression levels of macrophages cultured on fibrous or globular collagen. 142 proteins were found to be differentially expressed (Fig. 1c and Supplementary Table 2) and 101 of these proteins were more than 1.25-fold upregulated in macrophages cultured on fibrous collagen, whereas only 19 proteins were downregulated (Supplementary Table 2).

### Culturing macrophages on collagen results in higher expression of proteins involved in glycolysis

Out of the 77 differentially expressed proteins between macrophages cultured on collagen type I and uncoated controls, 30 proteins showed a more than twofold higher expression in collagen-coated conditions (Table 1). Three proteins even increased more than tenfold: pigment epithelium-derived factor (Serpfin1), protein FAM118B (Fam118B), and guanylate-binding protein 4 (GBP4). Only the proteins caspase-9 (Casp9), Rac GTPase-activating protein 1 (Racgap1), and SOSS complex subunit B1 (Nabp2) were expressed at lower levels in macrophages cultured on collagen-coated samples (Table 1).

**Table 1:** Effect of collagen type I on macrophage protein expression.

Gene name	Protein name	Fold change	p-value
Serpinf1	Pigment epithelium-derived factor	35.82	4.74E−03
Fam118b	Protein FAM118B	13.21	2.45E−02
Gbp4	Guanylate-binding protein 4	10.34	6.90E−03
Ndrg1	Protein NDRG1	6.22	4.74E−03
Syap1	Synapse-associated protein 1	6.02	2.45E−02
Nudt9	ADP-ribose pyrophosphatase, mitochondrial	5.95	1.35E−02
Golt1b	Vesicle transport protein GOT1B	5.27	2.05E−03
Sptlc1	Serine palmitoyltransferase 1	5.18	3.23E−03
Arfgap3	ADP-ribosylation factor GTPase-activating protein 3	5.02	6.90E−03
Thop1	Thimet oligopeptidase	4.93	9.70E−03
Slain2	SLAIN motif-containing protein 2	4.49	6.90E−03
Ethe1	Persulfide dioxygenase ETHE1, mitochondrial	4.14	1.35E−02
Mrps14	28S ribosomal protein S14, mitochondrial	3.95	1.82E−02
Trmt2a	tRNA (uracil-5-)-methyltransferase homolog A	3.37	3.23E−03
Eml2	Echinoderm microtubule-associated protein-like 2	3.19	6.90E−03
Alox5	Polyunsaturated fatty acid 5-lipoxygenase	3.19	4.74E−03
Ftl1	Ferritin light chain 1	3.11	1.82E−02
Ctnnd1	Catenin delta-1	2.52	1.82E−02
Nelfa	Negative elongation factor A	2.45	9.70E−03
Pfkl	ATP-dependent 6-phosphofructokinase, liver type	2.38	9.70E−03
Esyt2	Extended synaptotagmin-2	2.35	6.90E−03
Grhpr	Glyoxylate reductase/hydroxypyruvate reductase	2.34	3.23E−03
Acad8	Isobutyryl-CoA dehydrogenase, mitochondrial	2.26	2.45E−02
Tm9sf3	Transmembrane 9 superfamily member 3	2.18	2.45E−02
Fam162a	Protein FAM162A	2.12	1.82E−02
Agpat4	1-acyl-sn-glycerol-3-phosphate acyltransferase delta	2.11	2.45E−02
Armc10	Armadillo repeat-containing protein 10	2.10	6.90E−03
F13a1	Coagulation factor XIII A chain	2.06	1.82E−02
Ero1a	ERO1-like protein alpha	2.04	9.70E−03
Lrp1	Low-density lipoprotein receptor-related protein 1	2.01	4.31E−04
Casp9	Caspase-9	0.46	1.35E−02
Racgap1	Rac GTPase-activating protein 1	0.38	1.29E−03
Nabp2	SOSS complex subunit B1	0.30	1.82E−02

30 proteins were expressed at levels more than twofold higher by macrophages cultured on collagen type I-coated plastic (fibrous and globular combined, n = 6 each), compared to macrophages cultured on uncoated plastic (n = 6). Three proteins were expressed at levels more than twofold lower (fold change < 0.5). p < 0.025 was considered significant.

**Figure 2:**
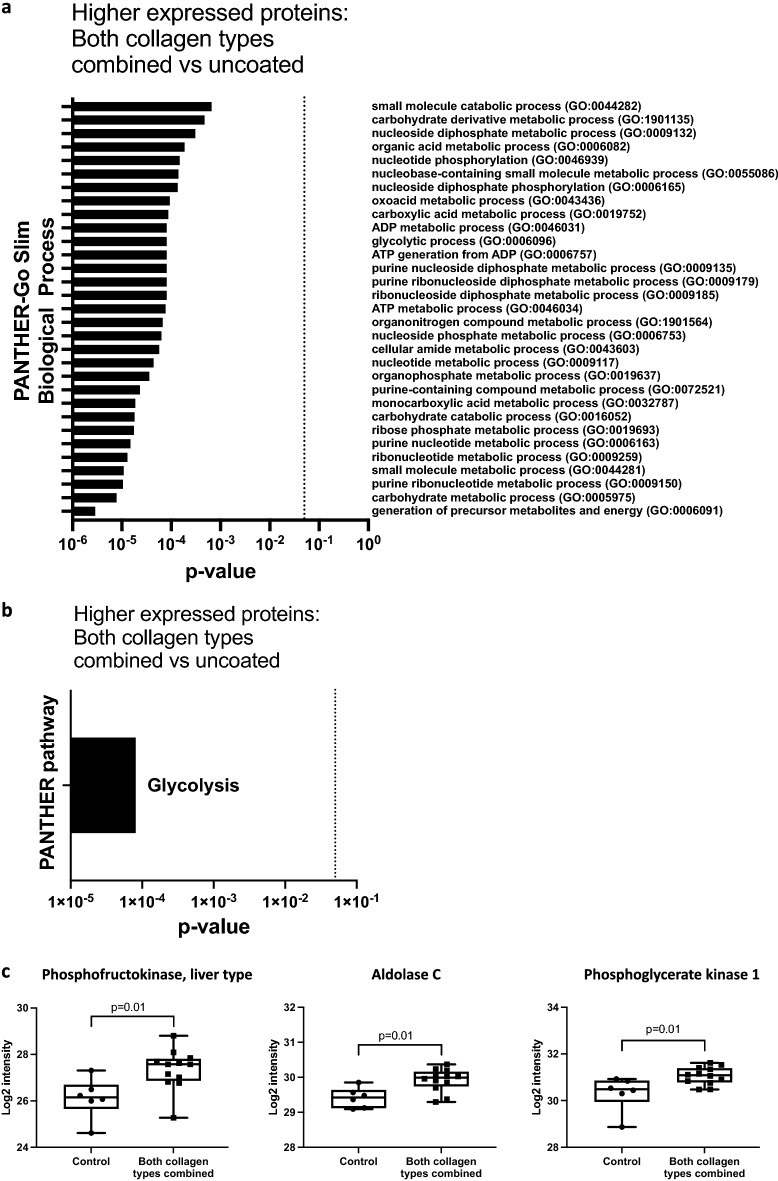
GO enrichment analysis of proteins upregulated by collagen type I. 69 proteins were > 1.25-fold upregulated by macrophages cultured on collagen-coated plates (fibrous and globular pooled), compared to uncoated controls. (**a**) PANTHER-Go Slim Biological Process analysis. (**b**) PANTHER pathway analysis, indicating significant enrichment of the glycolysis pathway. (**c**) Three proteins of the glycolysis pathway were significantly upregulated in macrophages grown on collagen (both types pooled, n = 6 each) compared to macrophages grown on plastic (control, n = 6). Protein intensity expressed as normalized Log2 values and statistically tested with an unpaired Wilcoxon test. p < 0.025 was considered significant. Data represented as a box and whiskers plot, where the box extends from the 25th to 75th percentiles and whiskers from min to max values.

**Figure 3:**
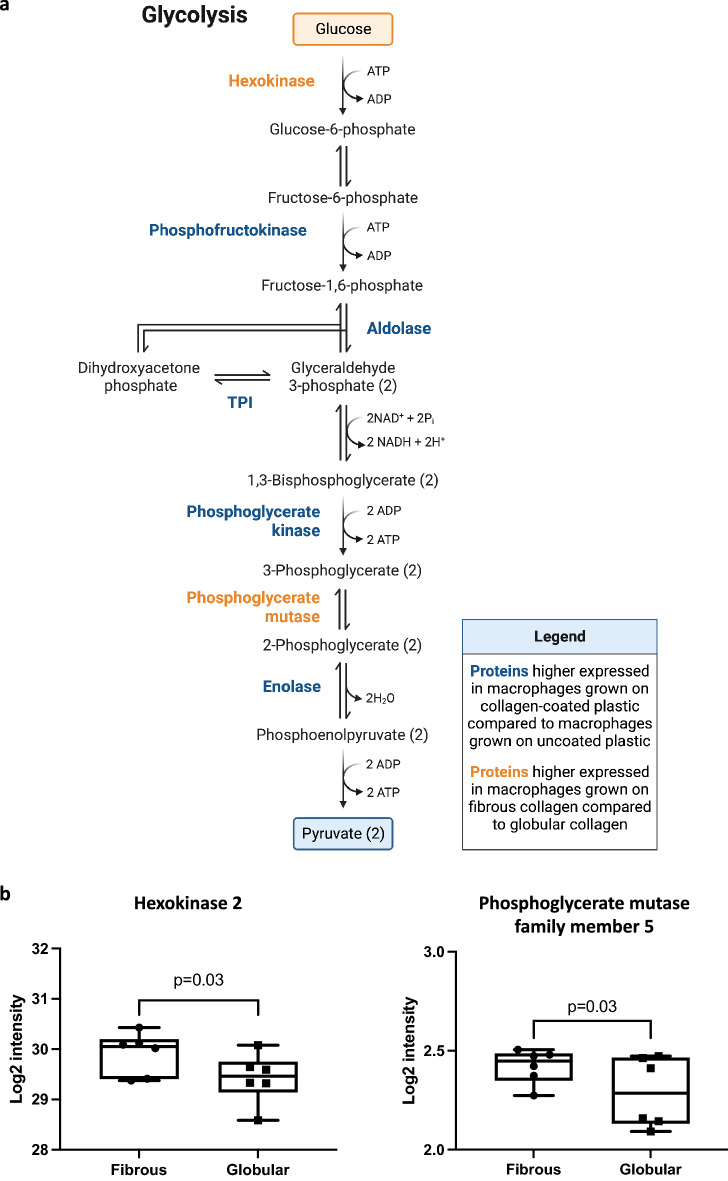
Glycolytic enzymes that were differentially expressed between macrophages cultured on fibrous or globular collagen. (**a**) Glycolysis metabolic pathway. Five proteins of the glycolysis pathway were significantly higher expressed in macrophages grown on collagen-coated plastic (fibrous and globular pooled, n = 6 each) compared to macrophages grown on uncoated plastic are indicated in blue. Proteins of the glycolysis pathway that were significantly higher expressed in macrophages grown on fibrous collagen compared to globular collagen are indicated in orange (fold change < 0.875 or > 1.25). (**b**) Two proteins of the glycolysis pathway were significantly higher expressed in macrophages grown on fibrous collagen compared to macrophages grown on globular collagen. Data are expressed as normalized Log2 values and statistically tested with a paired Wilcoxon test and p < 0.05 was considered significant (n = 6). Data represented as a box and whiskers plot, where the box extends from the 25th to 75th percentiles and whiskers from min to max values.

Analysis of the biological processes in which the proteins that are upregulated by culturing on collagen type I are involved (Supplementary Table 1), indicated an effect on macrophage metabolism (Fig. 2a). Pathway analysis revealed enrichment of the glycolysis pathway under collagen-coated conditions (Fig. 2b). The presence of either fibrous or globular collagen induced a more than twofold higher expression of liver type ATP-dependent 6-phosphofructokinase (Pfkl), a key enzyme in glycolysis. Additionally, the expression levels of two other members of the glycolysis pathway, fructose-biphosphate aldolase C (Aldoc) and phosphoglycerate kinase-1 (Pgk1) were higher in the presence of collagen type I (Fig. 2c). An overview of the main enzymes involved in the glycolysis pathway can be found in Fig. 3a.

### Higher expression of hexokinase 2 and phosphoglycerate mutase family member 5 by macrophages cultured on fibrous collagen as compared to globular collagen

Culturing macrophages on collagen type I clearly affected the expression of proteins. Next, we investigated if the morphology type of collagen would influence protein expression by comparing macrophages grown on fibrous collagen to macrophages cultured on globular collagen. Fibrous collagen led to a more than twofold higher expression of 23 proteins compared to globular collagen (Table 2), with the most pronounced fold-change observed for pigment epithelium-derived factor (Serpinf1). The expression of four proteins was found to be at least twofold lower in macrophages grown on fibrous collagen. These proteins are pyridoxal kinase (Pdxk), thioredoxin reductase 1, cytoplasmic (Txnrd1), nuclear cap-binding protein subunit 1 (Ncbp1) and translocating chain-associated membrane protein 1 (Tram1). Biological process and pathway analysis of the proteins with a > 1.25-fold change different expression (Supplementary Table 2) did not yield any significantly enriched pathways.

**Table 2:** Effect of collagen morphology on the protein expression profile of macrophages.

Gene name	Protein name	Fold change F/G	p-value
Serpinf1	Pigment epithelium-derived factor	12.66	3.13E−02
Dctn4	Dynactin subunit 4	9.97	3.13E−02
Rbm42	RNA-binding protein 42	9.86	3.13E−02
Mrps15	28S ribosomal protein S15, mitochondrial	9.52	3.13E−02
Atp7a	Copper-transporting ATPase 1	6.11	3.13E−02
Ppic	Peptidyl-prolyl cis–trans isomerase C	4.97	3.13E−02
Prim2	DNA primase large subunit	3.83	3.13E−02
Trim23	E3 ubiquitin-protein ligase TRIM23	3.64	3.13E−02
Mmp8	Neutrophil collagenase	3.63	3.13E−02
Uba5	Ubiquitin-like modifier-activating enzyme 5	3.40	3.13E−02
Plekha2	Pleckstrin homology domain-containing family A member 2	3.12	3.13E−02
Tmsb4x	Thymosin beta-4	3.09	3.13E−02
Unc45a	Protein unc-45 homolog A	2.61	3.13E−02
Ercc4	DNA repair endonuclease XPF	2.51	3.13E−02
Retreg3	Reticulophagy regulator 3	2.41	3.13E−02
Cybc1	Cytochrome b-245 chaperone 1	2.25	3.13E−02
Yipf4	Protein YIPF4	2.24	3.13E−02
Gdi1	Rab GDP dissociation inhibitor alpha	2.12	3.13E−02
Rdh11	Retinol dehydrogenase 11	2.11	3.13E−02
Rab11b	Ras-related protein Rab-11B	2.10	3.13E−02
Rtn3	Reticulon-3	2.05	3.13E−02
Wtap	Pre-mRNA-splicing regulator WTAP	2.05	3.13E−02
Apoo	MICOS complex subunit Mic26 (Apolipoprotein O)	2.02	3.13E−02
Pdxk	Pyridoxal kinase	0.50	3.13E−02
Txnrd1	Thioredoxin reductase 1, cytoplasmic	0.44	3.13E−02
Ncbp1	Nuclear cap-binding protein subunit 1	0.41	3.13E−02
Tram1	Translocating chain-associated membrane protein 1	0.39	3.13E−02

23 proteins were expressed at levels more than twofold higher in macrophages cultured on fibrous collagen type I-coated tissue culture plastic compared to macrophages in globular collagen-coated conditions, while four proteins were expressed at levels more than twofold lower (fold change < 0.5). N = 6, p < 0.05 was considered significant.

However, as we found the glycolysis pathway significantly enriched in cells grown on any type of collagen, we specifically investigated important metabolic enzymes in the list of significant proteins (Supplementary Table 2) and found hexokinase 2 (Hk2) and phosphoglycerate mutase family member 5 (Pgam5) to be differentially expressed (Fig. 3b). Therefore, glycolysis may again be one of the biological processes affected by the culture conditions of macrophages (Fig. 3a). In addition, four proteins in the list are part of the mitochondrial respiratory chain (Cytochrome b-c1, part of the ubiquinol-cytochrome c complex, elongation factor-like GTPase 1, NADH dehydrogenase (ubiquinone), and V-type proton ATPase), possibly pointing at changes in oxidative phosphorylation.

### No functional metabolic differences between macrophages cultured on collagen- or uncoated plastic

**Figure 4:**
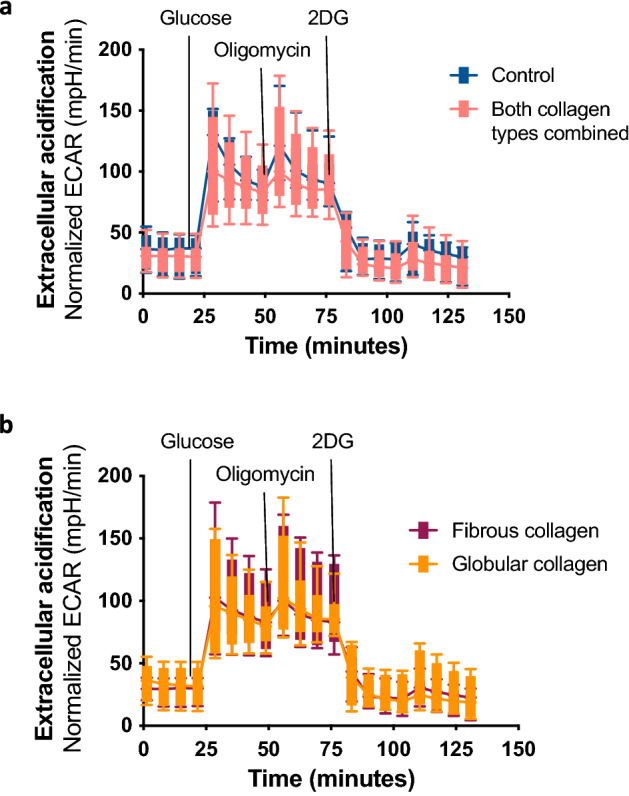
Extracellular acidification rate of alveolar macrophages cultured in collagen-coated or uncoated wells. After 72 h, extracellular acidification rate (ECAR) was measured by glycolysis stress test using an XFe96 extracellular flux analyzer and rates were normalized to protein concentrations. Macrophages were treated sequentially with glucose, oligomycin (ATP synthase inhibitor), and 2-DG (2-deoxyglucose). (**a**) Kinetic ECAR response of macrophages seeded in wells that were either coated with fibrous or globular collagen (data combined) or left uncoated (control). Each data point represents mean ± SD of 6 different experiments. Each single experiment had 6 technical replicates that were averaged. (**b**) Kinetic ECAR response of macrophages seeded in wells that were either coated with fibrous or globular collagen. Each data point represents mean ± SD of 6 different experiments. Each single experiment had 6 technical replicates that were averaged. Data represented as a box and whiskers plot, where the box and whiskers extend from the 10th to 90th percentile.

To investigate whether the higher expression of glycolytic proteins in macrophages cultured on collagen-coated substrates translated into changes in cellular metabolism, extracellular flux analysis was performed to assess glycolytic activity. No significant differences were found in glycolytic function between alveolar macrophages grown on collagen-coated or on uncoated wells (Fig. 4a). Similarly, no differences in glycolytic function were found between alveolar macrophages cultured on either fibrous or globular collagen (Fig. 4b). Furthermore, oxygen consumption rates were tracked and here too, no differences were found between macrophages cultured on plastic or macrophages cultured on either type of collagen (Supplementary Fig. 2).

**Figure 5:**
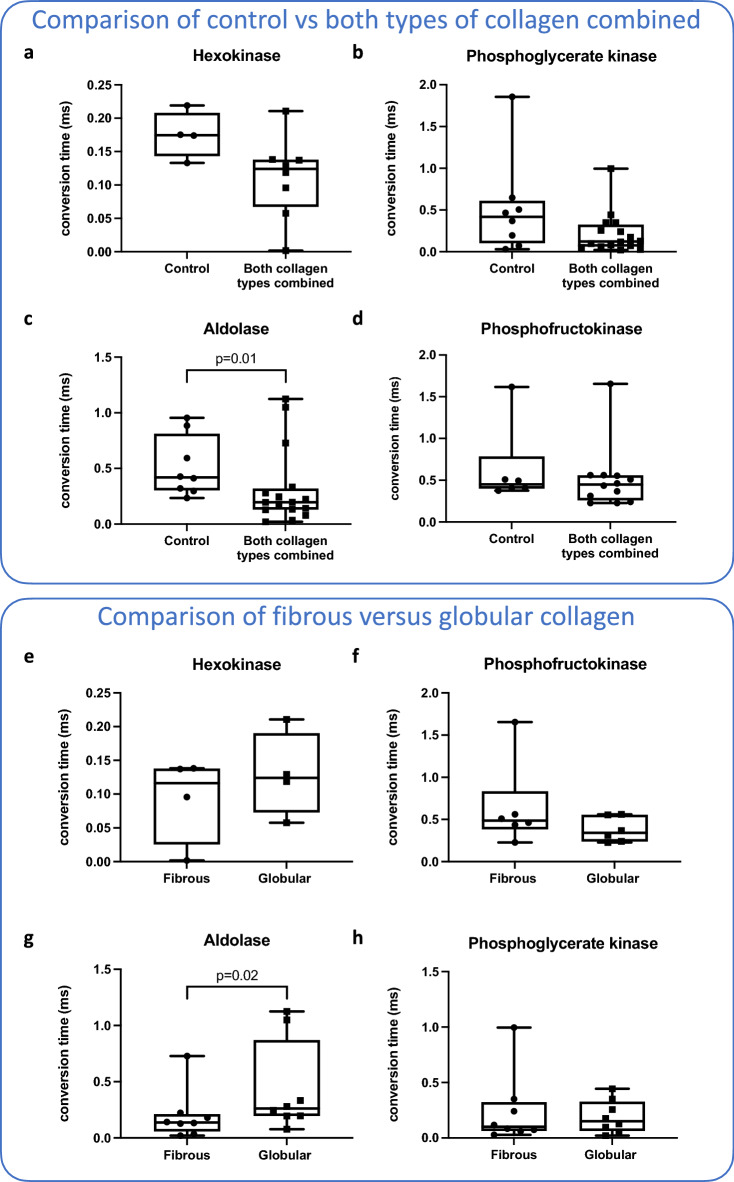
Activity of key glycolytic enzymes in alveolar macrophages in different culture conditions. Macrophages were cultured in wells coated with either fibrous or globular collagen or uncoated. After 72 h, enzymatic activity of four different glycolytic enzymes was measured in cell lysates. (**a**–**d**) Activity of glycolytic enzymes compared between macrophages cultured in uncoated and collagen type I-coated conditions (fibrous and globular pooled). Only the activity of aldolase was significantly lower in collagen-coated conditions. (**e**–**h**) Activity of glycolytic enzymes compared between macrophages cultured in fibrous collagen-coated and globular collagen-coated conditions. Only the activity of aldolase was significantly higher in globular collagen-coated conditions. Bars represent the mean rate of enzyme activity and each data point represents a different biological replicate (n = 4 for hexokinase, n = 6 for phosphofructokinase, and n = 8 for aldolase and phosphoglycerate kinase). Groups were compared using a Mann–Whitney U test (**a**–**d**) or Wilcoxon test (**e**–**h**). p < 0.05 was considered significant. Data represented as a box and whiskers plot, where the box extends from the 25th to 75th percentiles and whiskers from min to max values.

As we found no differences in glycolytic function, we investigated whether the activity of four of the differentially expressed glycolytic enzymes was affected by culture conditions, i.e. hexokinase, phosphofructokinase, aldolase, and phosphoglycerate kinase. First, we again compared macrophages cultured on any type of collagen versus those cultured on uncoated plastic. We found that all enzyme activities tended to be lower in macrophages cultured on collagen compared to the uncoated control, but only the activity of aldolase was significantly lower (Fig. 5a–d). We then investigated the effect of the collagen morphology on the activity of glycolytic enzymes and found that macrophages grown on fibrous collagen had significantly higher aldolase activity than macrophages grown on globular collagen (Fig. 5e–h). Opposite trends between protein expression and protein activity were observed: when protein expression was higher, the activity was lower and vice versa (compare Figs. 2c, 3b, 5a–h).

To investigate if there were any changes to proteins involved in oxidative phosphorylation, we specifically investigated the 33 proteins found in our data involved in the electron transport chain in mitochondria. We found only three proteins higher expressed in macrophages cultured on either globular or fibrous collagen compared to plastic (Cytochrome c oxidase subunit 4, mitochondrial, NADH: Ubiquinone Oxidoreductase Subunit B9, Ubiquinol-Cytochrome C Reductase Core Protein 1) and three lower expressed in macrophages grown on globular collagen compared to fibrous (Cytochrome C Oxidase Subunit 7A2, Superoxide Dismutase 2, Ubiquinol-Cytochrome C Reductase Complex III Subunit VII). Graphs of these six proteins can be found in Supplementary Fig. 3. The limited number of differentially expressed protein explains why the process of oxidative phosphorylation was not found enriched.

## Discussion

In this study, we gained insight into the effects of collagen type I morphologies on the proteomic signature of fetal liver-derived alveolar macrophages. Biological process and pathway enrichment analysis revealed clear effects of collagen on the expression of proteins involved in macrophage metabolism. Macrophages grown on collagen favor the expression of proteins involved in glycolysis, although this did not lead to increased glycolytic capacity, possibly because of a compensatory decrease in activity of those proteins. In addition to changes related to the general presence of collagen, fibrous and globular collagen also showed to have morphology-specific effects on the proteomic profile with prominent induction of Serpinf1 and prominent inhibition of Nabp2 on fibrous compared to globular collagen as the most differentially expressed proteins.

Pathway enrichment analysis revealed that macrophages cultured on collagen expressed higher levels of proteins involved in glycolysis and this was independent of collagen morphology. The expression of ATP-dependent 6-phosphofructokinase, liver type (Pfkl), a rate-limiting enzyme in glycolysis, was more than twofold higher in macrophages cultured on collagen type I. In macrophages, glycolysis is generally associated with an activated, pro-inflammatory phenotype^13^. However, we previously described higher expression of the mannose receptor CD206 and lower expression of major histocompatibility complex II (MHCII) on macrophages cultured on collagen type I, suggesting a more anti-inflammatory/pro-repair phenotype in these alveolar macrophages^11^. This discrepancy may be explained by the recent finding that alveolar macrophages do not depend on glycolysis to produce pro-inflammatory mediators^14^. Furthermore, glycolysis has been associated with a profibrotic macrophage phenotype in bleomycin-induced pulmonary fibrosis in mice^15^. In our study, the higher levels of glycolytic enzymes in macrophages grown on collagen did not functionally translate into higher glycolytic activity, based on the extracellular acidification rate analysis. This may have been the result of a compensatory lower activity of those enzymes, as there appeared to be a negative correlation between enzyme expression and activity. However, for proper correlation analysis, both analysis of enzyme concentration and activity would need to be performed on the same sample, which was not the case in our study.

Explanations of why more protein expression did not translate into more enzymatic activity could be found in changes in protein folding, post-translational changes, regulatory changes^16^, or allosteric regulation. For instance, phosphofructokinase has been shown to be allosterically regulated by ATP, AMP, fructose 1,6-bisphosphate, fructose 2,6-biphosphate, and citrate^17^. Another possible explanation could be the contributions of different enzyme isoforms. All enzymes of interest have more than one isoform, while the enzymatic activity tests we performed are not isoform-specific. The proteome analysis, however, assessed each specific isoform. Therefore, any difference in activity may have been obscured by contributions of other isoforms. Importantly, our results highlight that using only one analytical approach could lead to biased results when studying metabolic behavior of macrophages. Multiple techniques should be used when possible to gain complementary information^18^.

Even though pathway analysis of differentially expressed proteins in macrophages on fibrous versus globular collagen did not highlight any specific metabolic pathways, four of those proteins are part of the mitochondrial respiratory chain. These were cytochrome b-c1, part of the ubiquinol-cytochrome c complex, elongation factor-like GTPase 1, NADH dehydrogenase (ubiquinone), and V-type proton ATPase. The mitochondrial respiratory chain is one of the main energy sources used by the cells and is composed of five different multi-subunit respiratory complexes. Increased oxidative phosphorylation and fatty acid oxidation are usually associated with an alternatively activated, anti-inflammatory phenotype^19^. This is in line with our previous finding demonstrating that Ym1 is increased in macrophages cultured on fibrous collagen^11^ and would further explain why we do not see any differences in the glycolytic function.

The most affected protein by collagen and its morphology was the anti-angiogenic glycoprotein pigment epithelium-derived factor (PEDF), encoded by the Serpinf1 gene. PEDF can be secreted by macrophages and is known to bind to collagen type I in a microstructure-dependent manner. Therefore, it is suggested to be involved in collagen fibril assembly^20^. Furthermore, treatment of macrophages with PEDF has been shown to increase macrophage migration^21^, which could explain the higher levels of transmigration in macrophages cultured on fibrous collagen that we found before^11^, as they also express higher levels of PEDF. Interestingly, in lung tissue of patients with IPF, more PEDF expression was found than in lung tissue of controls^22^. PEDF is suggested to have a protective effect in the pathogenesis of fibrosis^23^ and therefore the higher levels found in fibrosis suggest a compensatory mechanism trying to control fibrosis development. Indeed, two studies have shown that increased expression of PEDF in macrophages attenuated collagen synthesis by fibroblasts in a macrophage-mediated manner^24,25^. This ties in with recent findings of downregulation of PEDF in acute exacerbations of IPF compared to stable disease as these exacerbations predispose to rapid progression of fibrosis^26^.

In addition to PEDF, the proteins dynactin subunit 4, RNA-binding protein 42, 28S ribosomal protein S15, and copper-transporting ATPase 1 were also expressed significantly more by macrophages on fibrous collagen than on globular collagen. No relevant data were found connecting dynactin subunit 4, RNA-binding protein 42, or 28S ribosomal protein S15 with macrophage function or lung fibrosis. However, the copper-transporting ATPase 1 (ATP7A) protein is of particular interest, since it has been suggested to play an important role in macrophage wound healing responses. In fact, ATP7A-downregulation inhibited macrophage infiltration in a mouse model for dermal wounding^27^. The higher levels of ATP7A may therefore also be linked to our previous results showing more migration in macrophages cultured on fibrous collagen^11^. Furthermore, ATP7A was shown to be required for copper delivery to members of the lysyl oxidase (LOX) family^28^. These copper-dependent enzymes play an important role in collagen cross-linking and higher activity of these enzymes has been described in patients with IPF^3,29,30^. Higher expression of ATP7A may therefore contribute to remodeling of non-organized fibrous collagen by activating members of the LOX family.

Only four proteins were expressed more than twofold higher by macrophages cultured on globular collagen than on fibrous collagen. Thioredoxin reductase-1 (Txnrd1) is an important component of the thioredoxin system, in which it reduces and thereby activates thioredoxin by transferring electrons from NADPH. This thioredoxin system has both anti-oxidative as well as anti-inflammatory properties and has been associated with antifibrotic effects in bleomycin-induced pulmonary fibrosis^31,32^. Cotreatment of macrophages with IL4 and recombinant thioredoxin promoted the development of an anti-inflammatory phenotype characterized by higher expression of CD206^33^. It is therefore conceivable that the higher levels of CD206 we previously found in macrophages cultured on globular collagen^11^ are related to the higher levels of Txnrd1. In addition to Txnrd1, three other proteins were expressed significantly more by macrophages on globular collagen than by macrophages on fibrous collagen. However, the role of these proteins remains to be elucidated as their direct function in macrophages is not yet clear. Of those three, only translocation-associated membrane protein-1 (Tram1) was found to have some connection with fibrosis. This protein was shown to be involved in translocation of proteins through the endoplasmic reticulum and in a study comparing patients with IPF and healthy controls, higher levels of TRAM1 mRNA were found in IPF^34^. The authors hypothesized that TRAM1 is upregulated because IPF is characterized by endoplasmic reticulum stress^34^.

Our study had some limitations. The number of proteins that was reproducibly quantified in all conditions was rather limited and only covered 17% (2.870 proteins) of the theoretical mouse proteome database (17.023 proteins). Consequently, GO enrichment analysis did not yield many hits. The absence of a cell cycle synchronization step may have contributed to this issue, but serum starvation is known to interfere with macrophage function and activation^35,36^ and was therefore intentionally omitted. As explained before, we tried to limit the variability of macrophage responses to collagen types by using fetal liver-derived macrophages. However, this does make translation to primary alveolar macrophages still an open question. Furthermore, the current study does not provide insight into how monocyte-derived macrophages would respond in comparison to fetal liver-derived alveolar macrophages, which is a critical area of investigation. It is well-documented that in cases of lung injury, monocyte-derived macrophages can replace fetal liver-derived alveolar macrophages in the lungs and that these two types of macrophages differ in their transcriptional and functional profiles^37,38^.

In conclusion, this study shows that the presence as well as the morphology of collagen type I have pronounced effects on the proteomic signature of alveolar macrophages. Collagen type I induced expression of proteins associated with macrophage metabolism, specifically glycolysis, while also collagen morphology had specific effects on the macrophage proteome. Although the studied collagen morphologies are not directly translatable to a healthy or fibrotic tissue in vivo, it does indicate that changes in collagen morphology can have a major impact on macrophage behavior. Several protein candidates have previously been described to play a role in processes related to fibrosis. Further verification and exploration of collagen type I-induced metabolic changes and morphology-dependent effects on the macrophage proteome will provide better insight into the mechanisms behind macrophage-matrix interactions and possibly yield new targets for the treatment of fibrosis.

## Materials and methods

### Substrate preparation

Sterile cell culture plates were coated with 75 µL/cm^2^ rat-tail collagen type I (Ibidi, Martinsried, Germany) at a concentration of 2.8 mg/mL at pH 3 (globular structured collagen layers) or pH 7 (fibrous collagen layers), as described before^11^. The desired pH was achieved by diluting the collagen type I stock in either 17.5 mM NaOH or 17.5 mM CH_3_COOH. Collagen layers were incubated at 37 °C for one hour, subsequently washed twice with sterile water and allowed to dry overnight at 37 °C.

### Cell culture

Alveolar macrophages derived from fetal monocytes according to the protocol of Fejer et al.^12^ (a kind gift by Dr. G. Fejer) were cultured in RPMI (Gibco Laboratories, Grand Island, USA) supplemented with 10% heat-inactivated FCS (Biowest, Nuaillè, France), 10 µg/mL gentamycin (Gibco Laboratories) and 20 ng/mL murine GM-CSF (Peprotech, Rocky Hill, USA). Once a week, macrophages were detached with 1 mM EDTA (Merck, Darmstadt, Germany) and reseeded at a density of 1 × 10^5^ cells/mL. The cells were incubated at 37 °C and 5% CO_2_, and cell culture medium was refreshed after 4 days. Macrophages between passage number 6 and 12 were seeded at a density of 5.7 × 10^4^ cells/cm^2^ for proteomic analysis or at a density of 35 × 10^4^ cells/cm^2^ for extracellular flux analysis.

### Sample preparation for proteomic analysis

Macrophages were cultured on collagen-coated or non-coated surfaces for 72 h before harvesting for proteomic analysis. Cell culture medium was collected and centrifuged to collect detached macrophages. The cell pellet was subsequently washed in PBS. Adherent macrophages were washed five times in PBS, followed by cell lysis with SDC lysis buffer: 1% w/v sodium deoxycholate (Sigma-Aldrich, Zwijndrecht, the Netherlands) and 1 mM EDTA in 0.1 M triethylammonium bicarbonate (TEAB) buffer (Thermo Fisher Scientific, Landsmeer, the Netherlands). The lysate of the adherent macrophages was then added to the pellet of detached macrophages and mixed. Subsequently, the total lysate was denatured at 98 °C for 5 min and sonicated with a tip sonicator (Vibra-Cell, Sonics, Newton, United States) for 3 × 10 s, 50% without pulse. Protein concentrations were determined with a microplate BCA protein assay following the manufacturer’s instructions (Pierce™ Microplate BCA Protein Assay Kit, Thermo Fisher Scientific). For tryptic in-solution digestion, 50 µg of the samples was diluted in 0.1 M TEAB (pH 8.3) to a final volume of 100 µL. For reduction, the samples were incubated with 10 mM dithiothreitol (DTT, dissolved in 0.1 M TEAB, pH 8.3) on a shaker (600 rpm) at 57 °C for 10 min. Afterwards, the samples were alkylated with 20 mM iodacetamide (0.1 M TEAB, pH 8.3) and incubated in the dark at room temperature for 30 min. Subsequently, free iodoacetamide was quenched by adding 10 mM of DTT (0.1 M TEAB, pH 8.3). For tryptic digestion, the samples were incubated in 1 µL trypsin solution (0.5 µg/µL sequencing grade modified trypsin, dissolved in trypsin resuspension buffer (Promega, Walldorf, Germany) at 37 °C for 16 h. Afterwards, the samples were acidified by adding formic acid to a final concentration of 1% and centrifuged at 16,000*g* at room temperature for 5 min. The supernatants were then transferred to a new reaction vial and evaporated to dryness.

### Liquid chromatography (LC)-mass spectrometry (MS)/MS analysis in data-dependent acquisition mode

For data independent analysis (DIA), a macrophage-specific protein/peptide library of the alveolar macrophages was generated using data dependent acquisition (DDA) mode. For this purpose, 100 µg of a dried tryptic digest of an alveolar macrophage reference sample was separated by high pH reversed-phase chromatography using a Dionex Ultimate 3000 ultra-performance liquid chromatography (UPLC) system equipped with a fraction collector. Peptides were separated with a C18 column (ACQUITY UPLC BEH C18 Column, 130 Å, 1.7 µm, 1 mm × 150 mm, Waters, Manchester, UK, buffer A: 5% acetonitrile (ACN), dissolved in HPLC-H_2_0, pH 9.5 adjusted with NH_4_OH; buffer B: 95% ACN, dissolved in HPLC-H_2_0, pH 9.5 adjusted with NH_4_OH) using a gradient from 1 to 50% buffer B in 60 min and a flow-rate of 100 µL/min. 30 fractions were collected with a volume of 200 µL. The fractions were pooled into 13 fractions and evaporated to dryness.

For LC–MS/MS analysis in data-dependent acquisition mode, the samples were dissolved in 20 µL of 0.1% formic acid and 1 µL was injected into a nano-ultra pressure liquid chromatography system (Dionex UltiMate 3000 RSLCnano pro flow, Thermo Fisher Scientific, Bremen, Germany) coupled via electrospray-ionization source to a tribrid orbitrap mass spectrometer (Orbitrap Fusion Lumos, Thermo Fisher Scientific, San Jose, CA, USA). The samples were loaded (15 µL/min) on a trapping column (nanoE MZ Sym C18, 5 μm, 180 µm × 20 mm, Waters, Eschborn, Germany, buffer A: 0.1% formic acid in HPLC-H_2_O; buffer B: 80% acetonitrile, 0.1% formic acid in HPLC-H_2_O) with 5% buffer B. After sample loading, the trapping column was washed with 5% buffer B for 2 min (15 μL/min) and the peptides were eluted (250 nL/min) onto the separation column (nanoE MZ PST CSH C18, 130 Å, 1.7 μm, 75 μm × 250 mm, Waters) and separated with a gradient of 5–37.5% B in 90 min. The spray was generated from a steel emitter (Thermo Fisher Scientific, Dreieich, Germany) at a capillary voltage of 1900 V. Full scan spectra were acquired over a m/z range from 400 to 1000 with a resolution of 60 k (at m/z 200) using a normalized automatic gain control target of 100% and a maximum injection time of 50 ms. Fragment spectra were acquired in data-dependent acquisition mode with a normalized high energy collision-induced dissociation energy of 28%, an orbitrap resolution of 30 k (at m/z 200), a normalized automatic gain control target of 100%, a maximum injection time of 100 ms and an intensity threshold for the precursor of 5e4. Fragment spectra were acquired in top-speed mode, with a full scan spectrum recorded every 3 s, and an exclusion time of 60 s.

Peptide and protein identification were performed with the Proteome Discoverer software suite (Thermo Fisher Scientific, version 2.4) using the Sequest search algorithm and PercolatorHT for false discovery rate calculation. The LC–MS/MS data were searched against the SwissProt mouse database (17.023 protein entries, UP000000589) and a contaminant database (235 entries) using the following parameters: precursor mass tolerance: 10 ppm, fragment mass tolerance: 0.02 Da, carbamidomethylation of cysteines was considered as a static modification, and the following modifications were considered as variable modifications: methionine oxidation, deamidation of glutamine and asparagine, Gln → pyro-Glu as N-terminal peptide modification, and an acetyl-loss, methionine-loss + Acetylation and a methionine-loss as N-terminal protein modification. Peptides and proteins were identified with a false discovery rate-cut-off of 0.01.

### LC-data independent acquisition (DIA)-MS analysis and data analysis

For DIA analysis, 1 µg of tryptic peptides was injected into a nano-ultra pressure liquid chromatography system and separated using the same parameters and conditions as described above (LC–MS/MS analysis in data-dependent acquisition mode). MS analysis was performed in DIA mode. Full scan spectra were acquired from 400 to 1000 with a resolution of 60 k (at m/z 200) using a normalized automatic gain control target of 100% and a maximum injection time of 50 ms. DIA fragment spectra were acquired with isolation windows of 10 m/z covering a m/z-range from 400 to 1000, a normalized high energy collision-induced dissociation energy of 28%, an orbitrap resolution of 30 k (at m/z 200), a normalized automatic gain control target of 100% and a maximum injection time of 54 ms. Fragment spectra were recorded over a m/z-range from 350 to 1500. One full spectrum was followed by a DIA scan from m/z 400–700, followed by a full spectrum and a DIA scan from m/z 700–1000.

For protein identification and quantification, a library was created with Skyline (MacCoss Lab, University of Washington, USA, version 19.1.0.193) based on the DDA data. The Proteome Discoverer results were imported in Skyline and the peptide library was generated using the following parameters: transition settings: precursor charges: 2, 3, 4, 5; fragment ion charges: 1, 2; ion types: p (precursor), b, y; ion match tolerance: 0.02 Da; minimum number of product ions: 4; m/z-range: 350–2000; the DIA acquisition method file was uploaded to determine the DIA isolation scheme; RT tolerance: 5 min. The generated library consisted of 6.310 proteins with at least one unique peptide for each protein and 74.654 unique peptides, covering 37% of the theoretical mouse proteome. The LC-DIA-MS files were imported and filtered with a dotp-threshold of 0.85 (best match). Only proteins with at least one unique peptide were kept. The Skyline results were exported for further data analysis in R and Python. This included missing values imputations, median normalization and log2 transformation of the protein intensities. For feature selection, partial least squares discriminant analysis and statistical analysis were performed.

### Extracellular flux analysis

The glycolytic rate was measured using an XFe96 Extracellular Flux Analyzer (Agilent Technologies, Santa Clara, California). Alveolar macrophages were seeded into Seahorse XF96 cell culture microplates that were coated with either fibrous or globular collagen or left uncoated (control) and incubated for 72 h. To measure extracellular acidification and oxygen consumption rates, cells were equilibrated for 1 h in the absence of CO_2_ at 37 °C in XF base media (Agilent Technologies, Santa Clara, California). The manufacturer’s protocol for the XF Glycolysis Stress Test was used to assess the glycolytic rate (Agilent Technologies). After equilibration, macrophages were stimulated with sequential injections of glucose (20 mM), oligomycin (1 µM), and 2-deoxy-d-glucose (50 mM). Glycolytic function is represented as glycolysis, glycolytic capacity and glycolytic reserve. Glycolysis was calculated as the difference between the maximal rate before oligomycin addition and the last rate measurement before glucose addition. Glycolytic capacity was calculated as the difference between the maximal rate after oligomycin addition and the last rate measurement before glucose addition. The glycolytic reserve was calculated as the difference between glycolytic capacity and glycolysis. Measurements were normalized to the protein amount in each well using a microplate BCA protein assay.

### Glycolytic enzyme kinetics

Glycolytic enzyme-catalyzed reactions were measured by means of photometric assays. Alveolar macrophages were seeded on plates coated with either fibrous or globular collagen, or left uncoated (control) and incubated for 72 h. Adherent cells were then detached with 1 mM EDTA, centrifuged, and washed twice with ice-cold PBS. Cells were then resuspended in PBS containing protease inhibitors (Sigma-Aldrich, cOmplete™ Protease Inhibitor Cocktail) and stored at − 80 °C. On the day of analysis, Triton X-100 (Sigma-Aldrich) was added to a final concentration of 0.1% and the suspension was centrifuged at 21,000*g* for 10 min at 4 °C. The cell-free supernatant was collected and used for the analysis. The enzyme activity, expressed in µmol/min/mg protein was determined from the difference in the slope of NAD(P)H absorbance (340 nm) before and after the addition of a substrate. The activities, unless stated otherwise, were measured in buffers with 5 mM KCl, 1 M Tris–HCl, 0.15 M NaCl, 5 mM CaCl_2_, and 0.1 M MgSO_4_, pH 7.4, 37 °C. The contents of the reaction mixtures were as follows: the phosphofructokinase (PFK) reaction mixture contained 0.1 M ATP, 15 mM NADH, 620 U/mL glycerol-3-phosphate dehydrogenase (G3PDH), 330 U/mL aldolase, 1800 U/mL triosephosphate isomerase (TPI), and 0.1 M fructose-6-phosphate. The phosphoglycerate kinase mixture contained 0.1 M ATP, 15 mM NADH, 800 U/mL glyceraldehyde-3P-dehydrogenase (GAPDH), and 0.125 M 3-phosphoglyceric acid. The aldolase mixture contained 15 mM NADH, 620 U/mL G3PDH, 1800 U/mL TPI, and 0.02 M of fructose-1,6-bisphosphate (F-1,6-BP). The pyruvate kinase mixture contained 0.1 M ADP, 0.02 M of F-1,6-BP, 15 mM NADH, 9300 U/mL lactate dehydrogenase, and 0.02 M phosphoenolpyruvate.

### Statistical analysis

Differentially expressed proteins by cells growing on collagen were investigated by comparing macrophages grown on plastic with macrophages grown on any type of collagen (globular + fibrous) using an unpaired two-sided two-sample Wilcoxon test with a Benjamini–Hochberg correction for multiple testing. To uncover differentially expressed proteins induced by a specific type of collagen, we compared macrophages grown on globular collagen with macrophages grown on fibrous collagen using a paired two-sided two-sample Wilcoxon test with a Benjamini–Hochberg correction for multiple testing. A corrected *p*-value < 0.05 was considered significant. No proteins were found to be significantly differentially expressed when using this correction. For pathway analyses we therefore used the uncorrected *p*-values and included all proteins that had an uncorrected *p*-value < 0.025 (collagen versus control) or < 0.05 (fibrous versus globular) and a fold change < 0.875 or > 1.25. Analyses were performed using R (version 4.2.0 (2022-04-22)—“Vigorous Calisthenics”).

*Pathway analysis* Enrichment of biological processes and pathways was analyzed by performing GO enrichment analysis with the annotation data sets ‘Panther GO-Slim Biological Process’ and ‘Panther Pathways’, using a Fisher’s Exact test to determine the p-value with a Benjamini–Hochberg correction to compensate for the false discovery rate. Results with a corrected *p*-value < 0.05 were considered to be significant.

*All other statistics* Experimental groups for other parameters were compared using GraphPad Prism 8.0 (GraphPad Software, La Jolla, USA). Control macrophages cultured on plastic were compared to macrophages cultured on any type of collagen using a Mann–Whitney U test, while the effect of collagen morphology was investigated by comparing macrophages grown on fibrous or globular collagen using a Wilcoxon matched-pairs signed-rank test. A *p*-value < 0.05 was considered significant.

## Supplementary Information

Supplementary Information.

## Data Availability

The raw data supporting the conclusion of this article will be made available by the authors upon reasonable request, without undue reservation.
