# Supplementary material for: Collagen type I alters the proteomic signature of macrophages in a collagen morphology-dependent manner
Source: Sci Rep. 2023 Apr 6;13:5670. doi: 10.1038/s41598-023-32715-0 (PMC10079972; doi:10.1038/s41598-023-32715-0)
Supplement: Supplementary file 1 — Supplementary Information. [file 41598_2023_32715_MOESM1_ESM.docx]

**Supplementary information to:**

**Collagen Type I Alters the Proteomic Signature of Macrophages in a Collagen Morphology-Dependent Manner**

Gwenda F. Vasse^1,2,3,4,#,^*, Sara Russo^5,#^, Andrei Barcaru^6^, Asmaa A.A. Oun^3,7^, Amalia M. Dolga^3^, Patrick van Rijn^1,2^, Marcel Kwiatkowski^8^, Natalia Govorukhina^5^, Rainer Bischoff^5^, Barbro N. Melgert^3,4^

^1^ University of Groningen, University Medical Center Groningen, Biomedical Engineering Department-FB40, Groningen, The Netherlands.

^2^ University of Groningen, University Medical Center Groningen, W.J. Kolff Institute for Biomedical Engineering and Materials Science-FB41, Groningen, The Netherlands.

^3^ University of Groningen, Department of Molecular Pharmacology, Groningen Research Institute of Pharmacy, Groningen, The Netherlands.

^4^ University of Groningen, University Medical Center Groningen, Groningen Research Institute for Asthma and COPD (GRIAC), Groningen, The Netherlands.

^5^ University of Groningen, Department of Analytical Biochemistry, Groningen Research Institute of Pharmacy, Groningen, The Netherlands.

^6^ University of Groningen, University Medical Center Groningen, Department of Laboratory Medicine, Groningen, The Netherlands.

^7^ Department of Cell Biochemistry, Groningen Institute of Biomolecular Sciences & Biotechnology, University of Groningen, Groningen, The Netherlands.

^8^ Functional Proteo-Metabolomics, Department of Biochemistry, University of Innsbruck, Innsbruck, Austria.

^#^ These authors contributed equally to this work.

* Corresponding author. E-mail address: g.f.vasse@rug.nl

**Supplementary figures**

**
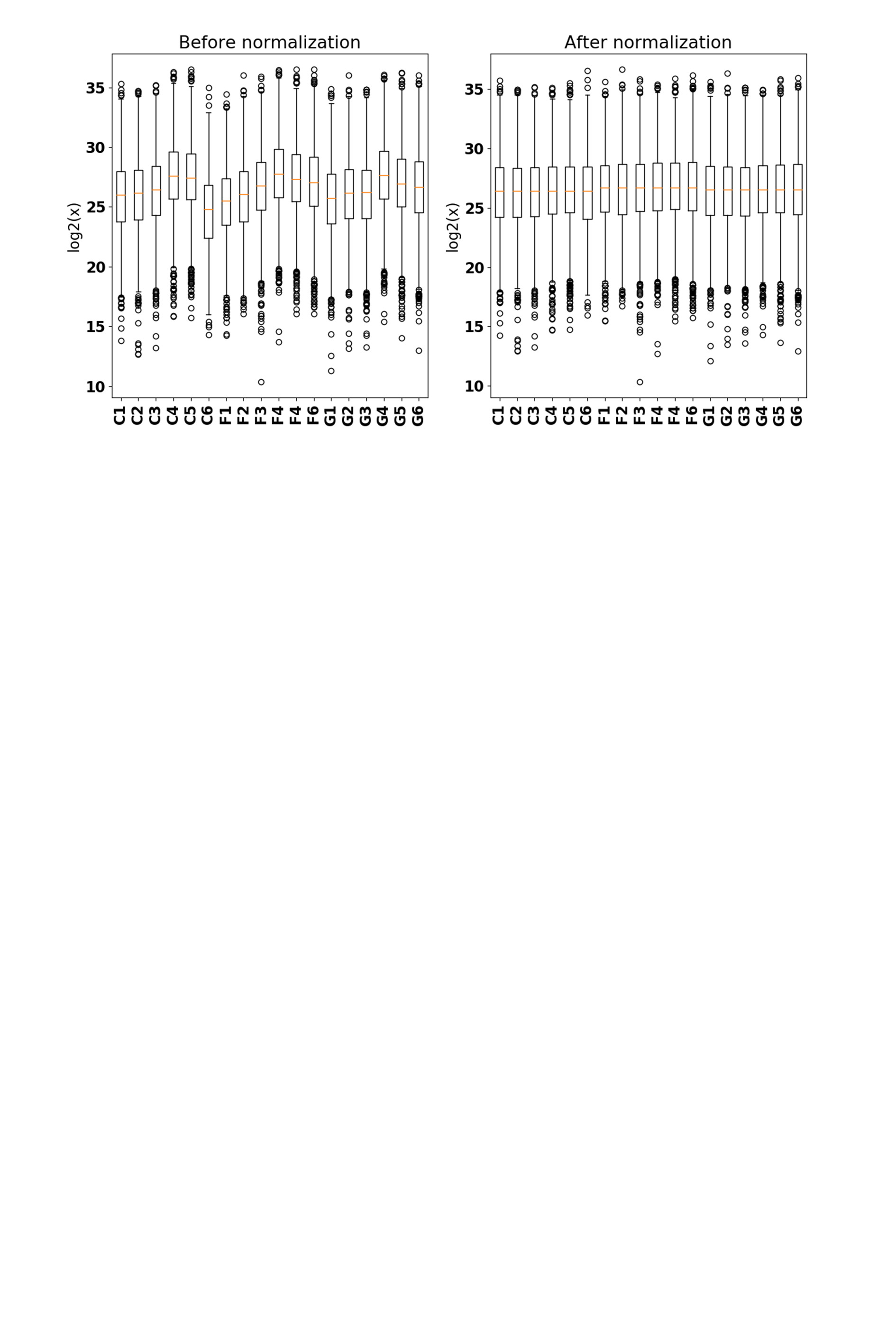
**

**Supplementary Fig. 1. Data normalization.** Log2 distributions of the proteins for each sample before and after preprocessing. Uncoated (C), fibrous collagen type I (F) and globular collagen type I (G).

**Supplementary Fig. 2.** Oxygen consumption rate of alveolar macrophages cultured in collagen-coated or uncoated wells. After 72 hours, oxygen consumption rate (OCR) was measured during glycolysis stress test using an XFe96 extracellular flux analyzer and rates were normalized to protein concentrations. Macrophages were treated sequentially with glucose, oligomycin (ATP synthase inhibitor), and 2-DG (2-deoxyglucose). **a.** Kinetic OCR response of macrophages seeded in wells that were either coated with fibrous or globular collagen (data combined) or left uncoated (control). Each data point represents mean ± SD of 6 different experiments. Each single experiment had 6 technical replicates that were averaged. **b.** Kinetic OCR response of macrophages seeded in wells that were either coated with fibrous or globular collagen. Each data point represents mean ± SD of 6 different experiments. Each single experiment had 6 technical replicates that were averaged. Data represented as a box and whiskers plot, where the box and whiskers extend from the 10th to 90th percentile.

**Supplementary Fig. 3.** Proteins of the electron transport chain that were significantly differentially expressed in macrophages grown on plastics versus either fibrous or globular collagen or significantly differentially expressed in macrophages grown on globular collagen versus either fibrous collagen. Expressed as normalized Log2 values and statistically tested with a Mann Whitney U test (control versus both collagen types combined) or a paired Wilcoxon test (fibrous versus globular collagen) and p<0.05 was considered significant (n=6). Data represented as a box and whiskers plot, where the box extends from the 25th to 75th percentiles and whiskers from min to max values. Cox4:Cytochrome c oxidase subunit 4; Ndufb9; NADH: Ubiquinone Oxidoreductase Subunit B9; Uqcrc1: Ubiquinol-Cytochrome C Reductase Core Protein 1; Cox7a1: Cytochrome C Oxidase Subunit 7A2; Sod2: Superoxide Dismutase 2; Uqcrq: Ubiquinol-Cytochrome C Reductase Complex III Subunit VII.

**Supplementary tables**

**Supplementary table 1**. **Morphology-independent effects of collagen type I on macrophage protein expression.** 77 proteins were differentially expressed by macrophages cultured on collagen type I-coated plastic compared to the uncoated control. Statistically tested with an unpaired Wilcoxon test. p<0.025 was considered significant.

| Gene name | Protein name | Fold change | | p-value | |
| --- | --- | --- | --- | --- | --- |
| Serpinf1 | Pigment epithelium-derived factor | | 35.82 | | 4.74E-03 |
| Fam118b | Protein FAM118B | | 13.21 | | 2.45E-02 |
| Gbp4 | Guanylate-binding protein 4 | | 10.34 | | 6.90E-03 |
| Ndrg1 | Protein NDRG1 | | 6.22 | | 4.74E-03 |
| Syap1 | Synapse-associated protein 1 | | 6.02 | | 2.45E-02 |
| Nudt9 | ADP-ribose pyrophosphatase, mitochondrial | | 5.95 | | 1.35E-02 |
| Golt1b | Vesicle transport protein GOT1B | | 5.27 | | 2.05E-03 |
| Sptlc1 | Serine palmitoyltransferase 1 | | 5.18 | | 3.23E-03 |
| Arfgap3 | ADP-ribosylation factor GTPase-activating protein 3 | | 5.02 | | 6.90E-03 |
| Thop1 | Thimet oligopeptidase | | 4.93 | | 9.70E-03 |
| Slain2 | SLAIN motif-containing protein 2 | | 4.49 | | 6.90E-03 |
| Ethe1 | Persulfide dioxygenase ETHE1, mitochondrial | | 4.14 | | 1.35E-02 |
| Mrps14 | 28S ribosomal protein S14, mitochondrial | | 3.95 | | 1.82E-02 |
| Trmt2a | tRNA (uracil-5-)-methyltransferase homolog A | | 3.37 | | 3.23E-03 |
| Eml2 | Echinoderm microtubule-associated protein-like 2 | | 3.19 | | 6.90E-03 |
| Alox5 | Polyunsaturated fatty acid 5-lipoxygenase | | 3.19 | | 4.74E-03 |
| Ftl1 | Ferritin light chain 1 | | 3.11 | | 1.82E-02 |
| Ctnnd1 | Catenin delta-1 | | 2.52 | | 1.82E-02 |
| Nelfa | Negative elongation factor A | | 2.45 | | 9.70E-03 |
| Pfkl | ATP-dependent 6-phosphofructokinase, liver type | | 2.38 | | 9.70E-03 |
| Esyt2 | Extended synaptotagmin-2 | | 2.35 | | 6.90E-03 |
| Grhpr | Glyoxylate reductase/hydroxypyruvate reductase | | 2.34 | | 3.23E-03 |
| Acad8 | Isobutyryl-CoA dehydrogenase, mitochondrial | | 2.26 | | 2.45E-02 |
| Tm9sf3 | Transmembrane 9 superfamily member 3 | | 2.18 | | 2.45E-02 |
| Fam162a | Protein FAM162A | | 2.12 | | 1.82E-02 |
| Agpat4 | 1-acyl-sn-glycerol-3-phosphate acyltransferase delta | | 2.11 | | 2.45E-02 |
| Armc10 | Armadillo repeat-containing protein 10 | | 2.10 | | 6.90E-03 |
| F13a1 | Coagulation factor XIII A chain | | 2.06 | | 1.82E-02 |
| Ero1a | ERO1-like protein alpha | | 2.04 | | 9.70E-03 |
| Lrp1 | Prolow-density lipoprotein receptor-related protein 1 | | 2.01 | | 4.31E-04 |
| Nnt | NAD(P) transhydrogenase, mitochondrial | | 1.90 | | 1.82E-02 |
| Pcbd2 | Pterin-4-alpha-carbinolamine dehydratase 2 | | 1.90 | | 9.70E-03 |
| Spcs2 | Signal peptidase complex subunit 2 | | 1.89 | | 6.90E-03 |
| Dhrs7 | Dehydrogenase/reductase SDR family member 7 | | 1.86 | | 1.82E-02 |
| Afg3l2 | AFG3-like protein 2 | | 1.80 | | 2.45E-02 |
| Basp1 | Brain acid soluble protein 1 | | 1.77 | | 1.82E-02 |
| Acot9 | Acyl-coenzyme A thioesterase 9, mitochondrial | | 1.76 | | 1.35E-02 |
| Tpm4 | Tropomyosin alpha-4 chain | | 1.75 | | 1.35E-02 |
| Gsn | Gelsolin | | 1.67 | | 2.45E-02 |
| Pgm1 | Phosphoglucomutase-1 | | 1.67 | | 2.45E-02 |
| Bcl10 | B-cell lymphoma/leukemia 10 | | 1.65 | | 2.45E-02 |
| Actn4 | Alpha-actinin-4 | | 1.64 | | 2.45E-02 |
| Atp5f1e | ATP synthase subunit epsilon, mitochondrial | | 1.61 | | 9.70E-03 |
| Plaa | Phospholipase A-2-activating protein | | 1.60 | | 1.82E-02 |
| Igf2bp2 | Insulin-like growth factor 2 mRNA-binding protein 2 | | 1.54 | | 9.70E-03 |
| Tagln2 | Transgelin-2 | | 1.53 | | 1.82E-02 |
| Nomo1 | Nodal modulator 1 | | 1.53 | | 2.45E-02 |
| Impa2 | Inositol monophosphatase 2 | | 1.52 | | 9.70E-03 |
| Hmox2 | Heme oxygenase 2 | | 1.52 | | 3.23E-03 |
| Itga5 | Integrin alpha-5 | | 1.51 | | 6.90E-03 |
| Aldoc | Fructose-bisphosphate aldolase C | | 1.50 | | 9.70E-03 |
| Psmd1 | 26S proteasome non-ATPase regulatory subunit 1 | | 1.49 | | 1.35E-02 |
| Anxa2 | Annexin A2 | | 1.48 | | 2.15E-04 |
| Pgk1 | Phosphoglycerate kinase 1 | | 1.46 | | 1.35E-02 |
| Erp44 | Endoplasmic reticulum resident protein 44 | | 1.44 | | 1.35E-02 |
| Acly | ATP-citrate synthase | | 1.44 | | 9.70E-03 |
| Map4 | Microtubule-associated protein 4 | | 1.40 | | 9.70E-03 |
| Sumf1 | Formylglycine-generating enzyme | | 1.38 | | 2.45E-02 |
| Naa25 | N-alpha-acetyltransferase 25, NatB auxiliary subunit | | 1.37 | | 3.23E-03 |
| Shmt2 | Serine hydroxymethyltransferase, mitochondrial | | 1.36 | | 9.70E-03 |
| Pgd | 6-phosphogluconate dehydrogenase, decarboxylating | | 1.32 | | 2.45E-02 |
| Pin1 | Peptidyl-prolyl cis-trans isomerase NIMA-interacting 1 | | 1.31 | | 1.82E-02 |
| Ppp2r1a | Serine/threonine-protein phosphatase 2A 65 kDa regulatory subunit A alpha isoform | | 1.31 | | 1.82E-02 |
| Vat1 | Synaptic vesicle membrane protein VAT-1 homolog | | 1.30 | | 1.35E-02 |
| Pak2 | Serine/threonine-protein kinase PAK 2 | | 1.29 | | 1.82E-02 |
| Itgal | Integrin alpha-L | | 1.28 | | 6.90E-03 |
| Cyb5r3 | NADH-cytochrome b5 reductase 3 | | 1.28 | | 1.82E-02 |
| Lmna | Prelamin-A/C | | 1.27 | | 1.35E-02 |
| Stx7 | Syntaxin-7 | | 1.25 | | 1.82E-02 |
| Hars1 | Histidine--tRNA ligase, cytoplasmic | | 1.22 | | 9.70E-03 |
| Gtf2f1 | General transcription factor IIF subunit 1 | | 1.21 | | 1.35E-02 |
| Cndp2 | Cytosolic non-specific dipeptidase | | 1.18 | | 1.35E-02 |
| Rpn1 | Dolichyl-diphosphooligosaccharide--protein glycosyltransferase subunit 1 | | 1.12 | | 2.45E-02 |
| Capzb | F-actin-capping protein subunit beta | | 1.08 | | 1.82E-02 |
|  |  | |  | |  |
| Casp9 | Caspase-9 | | 0.46 | | 1.35E-02 |
| Racgap1 | Rac GTPase-activating protein 1 | | 0.38 | | 1.29E-03 |
| Nabp2 | SOSS complex subunit B1 | | 0.30 | | 1.82E-02 |

**Supplementary table 2**. **Collagen morphology-specific upregulation of protein expression.** 142 proteins were differentially expressed by macrophages cultured on fibrous collagen type I-coated tissue culture plastic compared to macrophages in globular collagen-coated conditions. Statistically tested with a paired Wilcoxon test. p<0.05 was considered significant**.**

| Gene name | Protein name | Fold change F/G | p-value |
| --- | --- | --- | --- |
| **Serpinf1** | Pigment epithelium-derived factor | 12.66 | 3.13E-02 |
| **Dctn4** | Dynactin subunit 4 | 9.97 | 3.13E-02 |
| **Rbm42** | RNA-binding protein 42 | 9.86 | 3.13E-02 |
| **Mrps15** | 28S ribosomal protein S15, mitochondrial | 9.52 | 3.13E-02 |
| **Atp7a** | Copper-transporting ATPase 1 | 6.11 | 3.13E-02 |
| **Ppic** | Peptidyl-prolyl cis-trans isomerase C | 4.97 | 3.13E-02 |
| **Prim2** | DNA primase large subunit | 3.83 | 3.13E-02 |
| **Trim23** | E3 ubiquitin-protein ligase TRIM23 | 3.64 | 3.13E-02 |
| **Mmp8** | Neutrophil collagenase | 3.63 | 3.13E-02 |
| **Uba5** | Ubiquitin-like modifier-activating enzyme 5 | 3.40 | 3.13E-02 |
| **Plekha2** | Pleckstrin homology domain-containing family A member 2 | 3.12 | 3.13E-02 |
| **Tmsb4x** | Thymosin beta-4 | 3.09 | 3.13E-02 |
| **Unc45a** | Protein unc-45 homolog A | 2.61 | 3.13E-02 |
| **Ercc4** | DNA repair endonuclease XPF | 2.51 | 3.13E-02 |
| **Retreg3** | Reticulophagy regulator 3 | 2.41 | 3.13E-02 |
| **Cybc1** | Cytochrome b-245 chaperone 1 | 2.25 | 3.13E-02 |
| **Yipf4** | Protein YIPF4 | 2.24 | 3.13E-02 |
| **Gdi1** | Rab GDP dissociation inhibitor alpha | 2.12 | 3.13E-02 |
| **Rdh11** | Retinol dehydrogenase 11 | 2.11 | 3.13E-02 |
| **Rab11b** | Ras-related protein Rab-11B | 2.10 | 3.13E-02 |
| **Rtn3** | Reticulon-3 | 2.05 | 3.13E-02 |
| **Wtap** | Pre-mRNA-splicing regulator WTAP | 2.05 | 3.13E-02 |
| **Apoo** | MICOS complex subunit Mic26 | 2.02 | 3.13E-02 |
| **Pgam5** | Serine/threonine-protein phosphatase PGAM5, mitochondrial | 2.00 | 3.13E-02 |
| **Gltp** | Glycolipid transfer protein | 1.97 | 3.13E-02 |
| **Pdhx** | Pyruvate dehydrogenase protein X component, mitochondrial | 1.92 | 3.13E-02 |
| **Fam177a1** | Protein FAM177A1 | 1.91 | 3.13E-02 |
| **Crebbp** | Histone lysine acetyltransferase CREBBP | 1.90 | 3.13E-02 |
| **Abcf3** | ATP-binding cassette sub-family F member 3 | 1.90 | 3.13E-02 |
| **Bod1l** | Biorientation of chromosomes in cell division protein 1-like 1 | 1.85 | 3.13E-02 |
| **Efl1** | Elongation factor-like GTPase 1 | 1.77 | 3.13E-02 |
| **Ftl1** | Ferritin light chain 1 | 1.76 | 3.13E-02 |
| **C1qbp** | Complement component 1 Q subcomponent-binding protein, mitochondrial | 1.76 | 3.13E-02 |
| **Ero1a** | ERO1-like protein alpha | 1.75 | 3.13E-02 |
| **Tyms** | Thymidylate synthase | 1.75 | 3.13E-02 |
| **Iqgap2** | Ras GTPase-activating-like protein IQGAP2 | 1.74 | 3.13E-02 |
| **Sdf2l1** | Stromal cell-derived factor 2-like protein 1 | 1.74 | 3.13E-02 |
| **Sel1l** | Protein sel-1 homolog 1 | 1.74 | 3.13E-02 |
| **Sar1b** | GTP-binding protein SAR1b | 1.72 | 3.13E-02 |
| **Rhoc** | Rho-related GTP-binding protein RhoC | 1.71 | 3.13E-02 |
| **Hgh1** | Protein HGH1 homolog | 1.70 | 3.13E-02 |
| **Itgam** | Integrin alpha-M | 1.70 | 3.13E-02 |
| **Rps27** | 40S ribosomal protein S27 | 1.69 | 3.13E-02 |
| **Aimp1** | Aminoacyl tRNA synthase complex-interacting multifunctional protein 1 | 1.68 | 3.13E-02 |
| **Phf8** | Histone lysine demethylase PHF8 | 1.66 | 3.13E-02 |
| **Acot9** | Acyl-coenzyme A thioesterase 9, mitochondrial | 1.61 | 3.13E-02 |
| **Ddx6** | Probable ATP-dependent RNA helicase DDX6 | 1.61 | 3.13E-02 |
| **Impa2** | Inositol monophosphatase 2 | 1.60 | 3.13E-02 |
| **Uqcrq** | Cytochrome b-c1 complex subunit 8 | 1.59 | 3.13E-02 |
| **Nup50** | Nuclear pore complex protein Nup50 | 1.57 | 3.13E-02 |
| **Chil3** | Chitinase-like protein 3 | 1.57 | 3.13E-02 |
| **Aup1** | Lipid droplet-regulating VLDL assembly factor AUP1 | 1.56 | 3.13E-02 |
| **Ctsb** | Cathepsin B | 1.55 | 3.13E-02 |
| **F13a1** | Coagulation factor XIII A chain | 1.55 | 3.13E-02 |
| **Pcbd2** | Pterin-4-alpha-carbinolamine dehydratase 2 | 1.54 | 3.13E-02 |
| **Ufsp2** | Ufm1-specific protease 2 | 1.53 | 3.13E-02 |
| **Actr1a** | Alpha-centractin | 1.52 | 3.13E-02 |
| **Ndufv1** | NADH dehydrogenase [ubiquinone] flavoprotein 1, mitochondrial | 1.51 | 3.13E-02 |
| **Niban2** | Protein Niban 2 | 1.50 | 3.13E-02 |
| **Tpr** | Nucleoprotein TPR | 1.50 | 3.13E-02 |
| **Ptgs1** | Prostaglandin G/H synthase 1 | 1.50 | 3.13E-02 |
| **Sh3pxd2b** | SH3 and PX domain-containing protein 2B | 1.48 | 3.13E-02 |
| **Xpnpep1** | Xaa-Pro aminopeptidase 1 | 1.47 | 3.13E-02 |
| **Dnajc2** | DnaJ homolog subfamily C member 2 | 1.45 | 3.13E-02 |
| **Canx** | Calnexin | 1.44 | 3.13E-02 |
| **Sod2** | Superoxide dismutase [Mn], mitochondrial | 1.43 | 3.13E-02 |
| **Nudt9** | ADP-ribose pyrophosphatase, mitochondrial | 1.43 | 3.13E-02 |
| **Srp9** | Signal recognition particle 9 kDa protein | 1.43 | 3.13E-02 |
| **Cand1** | Cullin-associated NEDD8-dissociated protein 1 | 1.42 | 3.13E-02 |
| **Rab5c** | Ras-related protein Rab-5C | 1.41 | 3.13E-02 |
| **Elp3** | Elongator complex protein 3 | 1.41 | 3.13E-02 |
| **Anxa6** | Annexin A6 | 1.40 | 3.13E-02 |
| **Npepps** | Puromycin-sensitive aminopeptidase | 1.40 | 3.13E-02 |
| **Spcs1** | Signal peptidase complex subunit 1 | 1.40 | 3.13E-02 |
| **Lsm7** | U6 snRNA-associated Sm-like protein LSm7 | 1.39 | 3.13E-02 |
| **Ak2** | Adenylate kinase 2, mitochondrial | 1.38 | 3.13E-02 |
| **Eif3h** | Eukaryotic translation initiation factor 3 subunit H | 1.38 | 3.13E-02 |
| **Trpv2** | Transient receptor potential cation channel subfamily V member 2 | 1.37 | 3.13E-02 |
| **Hk2** | Hexokinase-2 | 1.37 | 3.13E-02 |
| **Gsn** | Gelsolin | 1.37 | 3.13E-02 |
| **Tpt1** | Translationally-controlled tumor protein | 1.35 | 3.13E-02 |
| **Mthfd1** | C-1-tetrahydrofolate synthase, cytoplasmic | 1.35 | 3.13E-02 |
| **Itgb5** | Integrin beta-5 | 1.35 | 3.13E-02 |
| **Clta** | Clathrin light chain A | 1.35 | 3.13E-02 |
| **Tm9sf3** | Transmembrane 9 superfamily member 3 | 1.35 | 3.13E-02 |
| **Fkbp1a** | Peptidyl-prolyl cis-trans isomerase FKBP1A | 1.35 | 3.13E-02 |
| **Psmd1** | 26S proteasome non-ATPase regulatory subunit 1 | 1.33 | 3.13E-02 |
| **Rrp1b** | Ribosomal RNA processing protein 1 homolog B | 1.33 | 3.13E-02 |
| **Atp2a2** | Sarcoplasmic/endoplasmic reticulum calcium ATPase 2 | 1.32 | 3.13E-02 |
| **Yars1** | Tyrosine--tRNA ligase, cytoplasmic | 1.31 | 3.13E-02 |
| **Myof** | Myoferlin | 1.31 | 3.13E-02 |
| **Srsf9** | Serine/arginine-rich splicing factor 9 | 1.31 | 3.13E-02 |
| **Anxa2** | Annexin A2 | 1.30 | 3.13E-02 |
| **Arhgef18** | Rho guanine nucleotide exchange factor 18 | 1.30 | 3.13E-02 |
| **Arpc2** | Actin-related protein 2/3 complex subunit 2 | 1.29 | 3.13E-02 |
| **Arl3** | ADP-ribosylation factor-like protein 3 | 1.29 | 3.13E-02 |
| **Dnajb1** | DnaJ homolog subfamily B member 1 | 1.28 | 3.13E-02 |
| **Ctnnd1** | Catenin delta-1 | 1.28 | 3.13E-02 |
| **Atp6v0a1** | V-type proton ATPase 116 kDa subunit a 1 | 1.28 | 3.13E-02 |
| **Rab14** | Ras-related protein Rab-14 | 1.27 | 3.13E-02 |
| **Hprt1** | Hypoxanthine-guanine phosphoribosyltransferase | 1.27 | 3.13E-02 |
| **Prdx6** | Peroxiredoxin-6 | 1.25 | 3.13E-02 |
| **Tmx1** | Thioredoxin-related transmembrane protein 1 | 1.24 | 3.13E-02 |
| **Pstpip2** | Proline-serine-threonine phosphatase-interacting protein 2 | 1.23 | 3.13E-02 |
| **Glod4** | Glyoxalase domain-containing protein 4 | 1.23 | 3.13E-02 |
| **Rplp0** | 60S acidic ribosomal protein P0 | 1.23 | 3.13E-02 |
| **Ppp6c** | Serine/threonine-protein phosphatase 6 catalytic subunit | 1.22 | 3.13E-02 |
| **Ywhaq** | 14-3-3 protein theta | 1.21 | 3.13E-02 |
| **Rps26** | 40S ribosomal protein S26 | 1.21 | 3.13E-02 |
| **Dync1i2** | Cytoplasmic dynein 1 intermediate chain 2 | 1.21 | 3.13E-02 |
| **Ctps2** | CTP synthase 2 | 1.21 | 3.13E-02 |
| **Casp6** | Caspase-6 | 1.20 | 3.13E-02 |
| **Ik** | Protein Red | 1.19 | 3.13E-02 |
| **Tmco1** | Calcium load-activated calcium channel | 1.19 | 3.13E-02 |
| **Drap1** | Dr1-associated corepressor | 1.18 | 3.13E-02 |
| **Ptges3** | Prostaglandin E synthase 3 | 1.18 | 3.13E-02 |
| **Myo1g** | Unconventional myosin-Ig | 1.17 | 3.13E-02 |
| **Arl6ip1** | ADP-ribosylation factor-like protein 6-interacting protein 1 | 1.16 | 3.13E-02 |
| **Cox7a2** | Cytochrome c oxidase subunit 7A2, mitochondrial | 1.16 | 3.13E-02 |
| **Tmx3** | Protein disulfide-isomerase TMX3 | 1.15 | 3.13E-02 |
| **Fabp4** | Fatty acid-binding protein, adipocyte | 1.15 | 3.13E-02 |
| **Noc2l** | Nucleolar complex protein 2 homolog | 1.13 | 3.13E-02 |
| **Psmd5** | 26S proteasome non-ATPase regulatory subunit 5 | 1.09 | 3.13E-02 |
| **Inpp5f** | Phosphatidylinositide phosphatase SAC2 | 0.84 | 3.13E-02 |
| **Cul2** | Cullin-2 | 0.84 | 3.13E-02 |
| **Rfc4** | Replication factor C subunit 4 | 0.84 | 3.13E-02 |
| **Psme2** | Proteasome activator complex subunit 2 | 0.84 | 3.13E-02 |
| **Pld4** | 5'-3' exonuclease PLD4 | 0.81 | 3.13E-02 |
| **Parp9** | Protein mono-ADP-ribosyltransferase PARP9 | 0.80 | 3.13E-02 |
| **Rpl6** | 60S ribosomal protein L6 | 0.79 | 3.13E-02 |
| **Fkbp3** | Peptidyl-prolyl cis-trans isomerase FKBP3 | 0.77 | 3.13E-02 |
| **Hspbp1** | Hsp70-binding protein 1 | 0.76 | 3.13E-02 |
| **Thyn1** | Thymocyte nuclear protein 1 | 0.76 | 3.13E-02 |
| **Trappc12** | Trafficking protein particle complex subunit 12 | 0.57 | 3.13E-02 |
| **Fam3c** | Protein FAM3C | 0.55 | 3.13E-02 |
| **Uap1** | UDP-N-acetylhexosamine pyrophosphorylase | 0.51 | 3.13E-02 |
| **Plcb3** | 1-phosphatidylinositol 4,5-bisphosphate phosphodiesterase beta-3 | 0.50 | 3.13E-02 |
| **Enpp4** | Bis(5'-adenosyl)-triphosphatase enpp4) | 0.50 | 3.13E-02 |
| **Pdxk** | Pyridoxal kinase | 0.50 | 3.13E-02 |
| **Txnrd1** | Thioredoxin reductase 1, cytoplasmic | 0.44 | 3.13E-02 |
| **Ncbp1** | Nuclear cap-binding protein subunit 1 | 0.41 | 3.13E-02 |
| **Tram1** | Translocating chain-associated membrane protein 1 | 0.39 | 3.13E-02 |
